# Innovative L-band electron paramagnetic resonance investigation of solid-state pouch cell batteries

**DOI:** 10.5194/mr-6-113-2025

**Published:** 2025-04-17

**Authors:** Charles-Emmanuel Dutoit, Raffaella Soleti, Jean-Marie Doux, Vincent Pelé, Véronique Boireau, Christian Jordy, Simon Pondaven, Hervé Vezin

**Affiliations:** 1 Université Lille Nord de France, CNRS, UMR8516, LASIRE, 59655 Villeneuve-d'Ascq, France; 2 Centre de Résonance Magnétique Electronique pour les Matériaux et l'Energie, 59655 Villeneuve-d'Ascq, France; 3 Université d'Angers, Inserm UMR1307, CNRS, UMR6075, Nantes Université, CRCI2NA, 49000 Angers, France; 4 Université d'Angers, SFR ICAT, Plateforme RPE, 49000 Angers, France; 5 SAFT, Direction de la Recherche, 111 Boulevard Alfred Daney, 33074 Bordeaux, France; 6 TotalEnergies OneTech R&D, Centre de Recherche de Solaize (CRES), Chemin du Canal, BP 22, 69360 Solaize, France

## Abstract

Usually, conventional electron paramagnetic resonance (EPR) spectroscopy and imaging employ a microwave cavity operating at X-band, i.e., with an excitation frequency of around 9.6 GHz, and this remains the most popular mode for the magnetic characterization of lithium batteries to date. Here, we provide the first low-frequency EPR investigations with respect to monitoring the metallic lithium structures in solid-state pouch cell batteries. We show that L-band, i.e., a microwave frequency of around 1.01 GHz, is an invaluable method to probe the electrode components directly through a standard pouch cell using aluminum-laminated film for packaging without opening the battery. These results offer a new approach for monitoring the nucleation of micrometric and submicrometric lithium particles, such as dendritic lithium structures, and is an important step in the development of reliable solid-state batteries.

## 1 Introduction

Lithium-ion (Li-ion) batteries offer high energy densities, making them appealing for a wide range of applications. Conventional Li-ion cells are composed of a Li-containing oxide, as a positive electrode material, and graphite, as a negative electrode material. The Li^+^ ionic transfer between the electrodes is done through a liquid electrolyte flooding both electrodes and a separator keeping both electrodes apart [Bibr bib1.bibx2]. One of the attractive cathodes for commercial use is made up of a Li-containing metal oxide such as lithium–nickel–manganese–cobalt oxide, commonly known as NMC [Bibr bib1.bibx17]. These elements form a stable structure to hold the lithium ions when the battery is in a discharged state. With the goal of improving the energy density and safety of cells, the replacement of the liquid electrolyte by a solid compound (gel, polymer, ceramic, or their combination) is being studied in solid-state batteries [Bibr bib1.bibx1]. While most commercial Li-ion cells are hosted in rigid structures (cylindrical and prismatic cells), softer casing made of laminated aluminum and a polymer layer are also being developed for pouch cell packaging. This format can easily be made into different shapes and sizes, while benefiting from a lightweight packaging, which is a serious advantage for mobility applications. Owing to the lightweight aluminum packaging, pouch cells can be used in small portable electronics, such as drones and mobile phones. Compared with rigid cylindrical cells, they offer a good weight-to-energy ratio. Considering the versatility of the pouch cell format, it is also adapted for solid-state batteries. Furthermore, the packaging is not a rigid enclosure; therefore, special attention is required during short circuits or overcharge events, as the pressure buildup can cause cell swelling [Bibr bib1.bibx3]. Such short circuits, usually caused by the nucleation of metallic lithium aggregates during lithiation processes, can pose a serious risk of explosion. Thus, early nondestructive detection of lithium growth is mandatory to avoid such risks in this cell format.

In this work, using continuous-wave L-band electron paramagnetic resonance (cw-EPR) spectroscopy and imaging, we report, for the first time, the possibility of analyzing the metallic lithium structures in solid-state pouch cell batteries directly through standard aluminum-laminated film. One of limitations of the EPR characterization of an electrochemical cell is the cell design, which has to be compatible with the standard resonator dimension. In previous EPR measurements, we designed a millimetric cylindrical electrochemical cell specially adapted for X-band spectrometers [Bibr bib1.bibx16]. It should be noted that all previous in situ EPR results have been performed on electrochemical cell models using a Kapton film, PET (polyethylene terephthalate) or EVA (ethylene vinyl acetate) film, or other types of EPR-silent polymers that are not standard packaging for batteries [Bibr bib1.bibx9]. Herein, we demonstrate that the electrode materials enveloped in an Al-based pouch film, used as a standard packaging material for batteries [Bibr bib1.bibx15], can be characterized without opening the battery. This result offers a new approach that paves the way for operando characterization of commercial pouch cell batteries.

## 2 Experimental details

### 2.1 Samples

Two types of cells were assembled using the same positive electrode and separator layers with either a Li-metal or Si-based negative electrode. An argyrodite-type sulfide electrolyte was selected as the solid electrolyte (SE; Li_6_PS_5_Cl from NEI Corporation). The positive electrode, separator, and Si-based negative electrode were all prepared using a wet process employing isobutyl isobutylene and xylene solvents with PVdF-based copolymer (where PVdf denotes polyvinylidene fluoride) as the binder for the electrodes and a specific in-house binder for the separator. All of the materials were handled and processed in an Ar-filled glove box. For the positive electrode, NMC811 (LiNi_0.8_Mn_0.1_Co_0.1_O_2_, commonly used by cathode) and SE powders were dispersed in a binder gel solution using a planetary mixer. A slurry containing NMC811, SE, and PVdF was cast on a carbon-coated Al foil with a doctor blade with a 20.5 mg cm^−2^ loading. For the Si-based negative electrode, Si (micrometer size), SE, and Super P carbon were dispersed in the binder gel solution. A slurry containing Si, SE, C, and PVdF was cast on a carbon-coated Cu foil with a doctor blade, and a 2.9 mg cm^−2^ loading was obtained. For the separator, the SE powder was dispersed in the binder gel solution, and the obtained SE : binder (weight ratio 97:3 ) slurry was cast on a release-type PET film with a doctor blade. All of the layers were dried at room temperature in the glove box. The uncalendared separator had a thickness of 130 µ m ( ± 10 µ m) and could be peeled off the PET film to recover a self-standing solid electrolyte layer. The Li metal had a thickness of 60 µ m. The different layers were punched. The positive and negative electrodes were welded to tabs and set in the polypropylene-coated aluminum laminate before they were sealed and the pouch was obtained. The pouch, made of aluminum laminate (120 µ m) containing one polyamide layer and one polypropylene layer, was compacted at 300 MPa using a cold isostatic press to densify the layers and improve the electrode–separator contacts. The voltage was then controlled with a voltmeter, and it was ensured that the voltage was above 2 V, which indicated that the pouches had not short-circuited.

### 2.2 Electron paramagnetic resonance spectroscopy

Continuous-wave (cw) EPR experiments were performed using an L-band Bruker spectrometer operating at a microwave frequency of around 1.01 GHz. EPR measurements were made at room temperature in a cylindrical microwave loop-gap cavity produced by Bruker (L-band bridge loop-gap resonator, 36 mm E1978). The microwave power applied into the loop-gap cavity was set to 36 mW. The modulation amplitude of the magnetic field was taken at 0.3 mT. Other spectrometer settings were as follows: sweep width of 20 mT, conversion time of 40 ms, number of scans equal to 1, and sweep time of 40.96 s. Simulations were done using the EasySpin package for MATLAB [Bibr bib1.bibx19].

### 2.3 Electron paramagnetic resonance imaging

EPR imaging measurements were performed with an L-band Bruker imaging spectrometer equipped with a three-axis gradient coil set with gradients along the x axis (perpendicular to Y and B ) , y axis (perpendicular to X and B ), and z axis (along B ). Images were done with a gradient strength of 4.2 mT cm^−1^ and a field of view of 30 mm. A total of 157 projections were recorded for each image. The high-resolution images were reconstructed with a size of 200 pixels × 200 pixels, resulting in a pixel size of 150 µ m. The recorded projections under gradient were deconvoluted from a signal obtained without gradient. Finally, the spatial–spatial images were obtained after a filtered back-projection.

## 3 Results and discussion

Low-frequency EPR experiments were carried out to probe the electrode materials of two solid-state pouch cell batteries. In situ pouch cells were measured in their pristine state without opening the batteries. Figure [Fig Ch1.F1] shows the real dimensions of the NMC ∥ Li^0^ pouch cell battery and its orientation inside the loop-gap cavity. Usually, the z axis corresponds to the static magnetic field direction ( B ). The 65 mm × 38 mm pouch cells are placed in the microwave loop-gap cavity (diameter of ∼ 40 mm and length of ∼ 70 mm) such that the battery plane is oriented along the static magnetic field, i.e., perpendicular to the y axis. However, probing conduction electrons in metallic structures is not trivial due to the limited penetration of the microwave in the conductor, also known as the “skin depth”. Indeed, it is well known that the EPR line shape of metallic conductors is sensitive to the metal thickness and the skin depth. In an EPR experiment, the electromagnetic field penetrates only the skin depth $\delta_{\mathrm{mw}}$ , and only the spins located in this area are excited. Consequently, the line shape is influenced by the homogeneousness of the electromagnetic field in the metallic structure, displaying a Dysonian line shape when a fraction of the electronic spins experience the microwave field or a pure Lorentzian line shape in the opposite case (when all of the electronic spins are excited). When the metal thickness is much higher than the skin depth, the EPR spectrum exhibits an asymmetric (Dysonian) line shape characterized by the ratio A / B ≫ 1, resulting from the apparent amplitude ratio between its positive lobe ( A ) and negative lobe ( B ) [Bibr bib1.bibx10]. This line shape is also influenced by the spin–spin relaxation time $T_{2}$ and, thus, the linewidth, which depends on the metallic lithium morphology ( $T_{2}$ ∼ 10^−8^ s for bulk lithium or $T_{2}$ ∼ 10^−6^ s for micrometric lithium aggregates). Therefore, bulk lithium exhibits a linewidth much larger than the micrometric lithium structures. This skin depth can be easily calculated as follows:

1 $$\delta_{\mathrm{mw}}\propto \sqrt{\frac{\rho }{f}},$$

where ρ is the metallic resistivity and f is the microwave frequency applied inside the cavity. It can be seen that the skin depth is proportional to $1/\sqrt{f}$ . However, the physical size and the conductive behavior of the standard laminated aluminum packaging lead to an additional challenge with respect to the monitoring of electrode materials. Indeed, placing a large metallic conductor inside a standard X-band microwave cavity can cause serious perturbations of the resonator, making it impossible to tune the cavity. However, obtaining optimal tuning is essential to perform EPR measurements. Consequently, we turned to L-band EPR spectroscopy and imaging, which have a large loop-gap cavity adapted for standard solid-state pouch cell batteries (i.e., no specific battery preparation is needed for X-band). In such a loop-gap cavity, the electrical field E is confined to the capacitive gap and the magnetic field B inside the inductive loop. As a result, the microwave resonator is undisturbed by the insertion of the metallic sample (see Fig. [Fig Ch1.F1]b). Furthermore, at L-band, the skin depth increases compared with X-band, giving a better microwave penetration through the metallic body ( ∼ 3 µ m for aluminum at 1 GHz).

**Figure 1 Ch1.F1:**
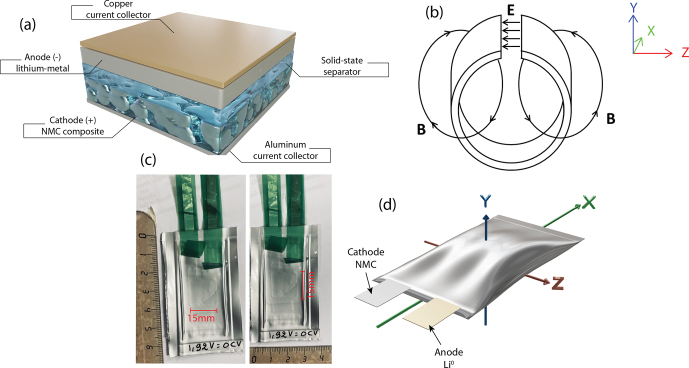
Examples of the pouch cells studied in this work. **(a)** Schematic representation of the stacking used in our solid-state battery NMC ∥ Li^0^. **(b)** Sketch of a microwave loop-gap cavity used for L-band EPR spectroscopy. The electrical field ( E ) is confined to the capacitive gap, whereas the magnetic field ( B ) is confined inside the inductive loop. **(c)** Digital photos of the NMC ∥ Li^0^ pouch cell. **(d)** Schematic representation of the pouch cell with its orientation inside the microwave loop-gap cavity.

Figure [Fig Ch1.F2]a gives the L-band EPR spectrum of the NMC ∥ Li^0^ pouch cell recorded at room temperature and using a continuous wave through the standard aluminum-laminated packaging. This signal exhibits a Dysonian line shape usually found for conduction electrons [Bibr bib1.bibx6]. To obtain more information about the nature of this metallic complex, like the g factor and the linewidth, we simulated the spectrum with a phase-shifted Lorentzian function, i.e., a sum of Lorentzian-shaped absorption and dispersion functions, modeling the Dysonian shape [Bibr bib1.bibx10]. It can be seen that the EPR spectrum is characterized by the following: (i) a single line centered at a g factor of around 2.004 ± 2.10^−3^, (ii) a peak-to-peak linewidth of 0.3 mT, and (iii) an asymmetric A / B ratio of around 3.7, as expected for metallic lithium structures. It is worth noting that the skin depth is around 4 µ m for Li^0^ complexes at 1 GHz. This result indicates that the metallic structure size detected is much higher than 4 µ m, which is consistent with the metallic electrode dimensions. As the solid-state NMC ∥ Li^0^ battery is composed of a metallic lithium electrode, we may compare the spectral signature relative to a nonmetallic battery taken as a reference. The result is given in Fig. [Fig Ch1.F2]b, where a lithium metal anode is replaced by a Si-based electrode. As expected, the solid-state NMC ∥ Si-based battery, which contains (EPR-silent) Li^+^, Ni^2+^ ( S=1 ), Ni^3+^ ( S=1/2 ), Mn^4+^ ( S=3/2 ), (diamagnetic) Co^3+^, and (EPR-silent) O^2−^, initially gives no metallic lithium spectrum in the pristine state. However, as reported in the literature [Bibr bib1.bibx20], at room temperature, NMC material exhibits an EPR signal centered at a g value of around 2.00 with a linewidth of around 22 mT arising from Mn^4+^, Ni^2+^, and Ni^3+^. The manifestation of the NMC is represented by a distortion of the baseline. This distortion is clearly visible in the NMC ∥ Si-based cell, but it is invisible in the NMC ∥ Li^0^ cell due to the intense Li^0^ signal. Nevertheless, a single EPR peak is observed, displaying a g factor near the free-electron g value ( $g_{e}=2.0023$ ), which originates from the SiO_2_ defects of the anode.

**Figure 2 Ch1.F2:**
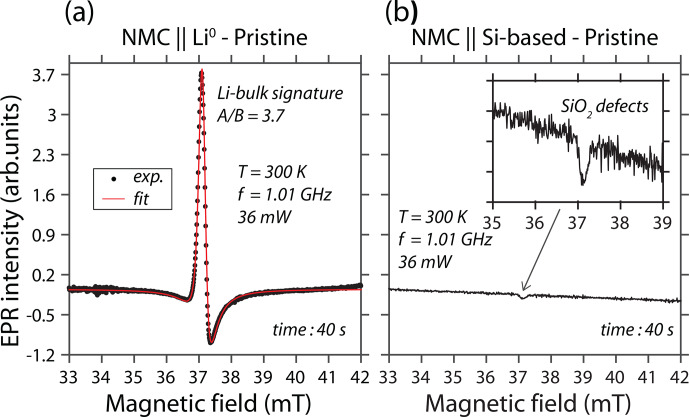
Continuous-wave L-band EPR spectra of two solid-state batteries recorded through the aluminum-laminated pouch cell at room temperature and in their pristine state: **(a)** NMC ∥ Li^0^ and **(b)** NMC ∥ Si-based battery. The modulation amplitude is 0.3 mT, the microwave power is 36 mW, and the microwave frequency is 1.01 GHz.

**Figure 3 Ch1.F3:**
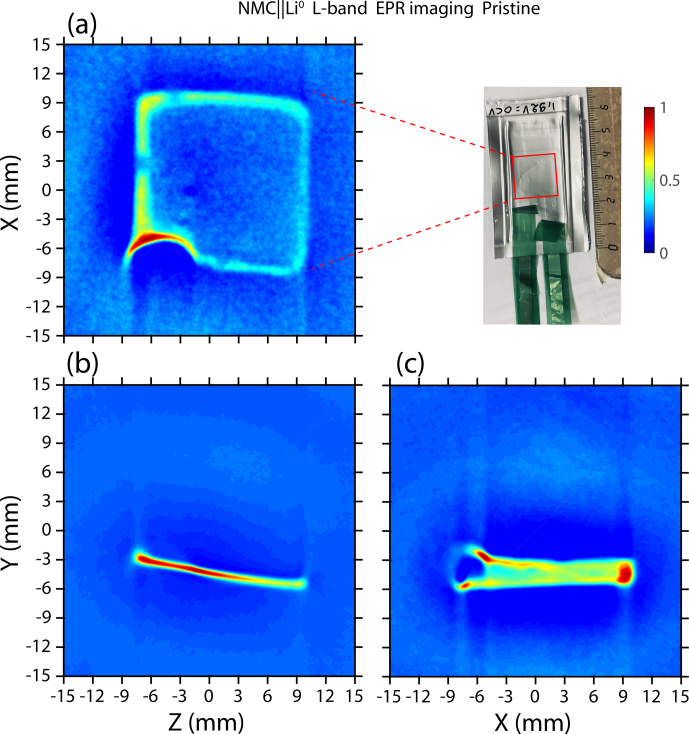
In situ L-band EPR imaging of a solid-state pouch cell NMC ∥ Li^0^ battery recorded using the spatial–spatial detection scheme in the **(a)** x – z plane, **(b)** y – z plane, and **(c)** y – x plane.

In order to verify that the metallic lithium spectrum does not originate from an impurity inside the pouch cell, we turned to the spatial–spatial imaging of the NMC ∥ Li^0^ solid-state battery to localize the anode part. As we saw previously, metallic lithium structures give a relatively sharp spectrum with a linewidth of around 0.3 mT. Using a gradient strength of 4.2 mT cm^−2^ and thanks to the sharp line of Li^0^, high-resolution EPR images in the respective x – z , y – z , and y – x spatial planes are expected. After placing the pouch cell directly in the center of the microwave loop-gap cavity and gradient coils, we recorded each image at room temperature. The time needed for each spatial–spatial image is around 60 min with a pixel size of 150 µ m and a field of view of 30 mm. Figure [Fig Ch1.F3] presents the EPR images for an NMC ∥ Li^0^ solid-state pouch cell battery. For more clarity, we chose to reconstruct the spin concentration using a contour map, with the red contour showing the highest amplitude and the blue contour showing the lowest amplitude. Contrary to recent investigations using X-band EPR imaging to monitor electrochemical batteries with packaging made with a nonstandard material, such as a Kapton film [Bibr bib1.bibx9], the L-band image contains the spin distribution of electrode materials measured directly through the standard aluminum-laminated pouch cell. The Li^0^ anode part of the pouch cell is clearly visible and appears as a (15 mm × 15 mm) square shape in the center of image. Within the limit of the resolution of an L-band EPR spectrometer, this shape and its dimensions are similar to the real size of the anode. However, variation in the EPR intensities is observed between the edge and the middle of the anode. As shown by [Bibr bib1.bibx14], the EPR signal of the thick and flat metallic electrode (much thicker than the skin depth) gives a higher apparent intensity at the edge, which hides the weaker signal in the middle part of the lithium foil. The physical origin of this contrast, characterized by extreme sensitivity of the edge to the microwave field, comes from a local variation in the microwave field caused by shielding and/or eddy current effects [Bibr bib1.bibx14]. Nevertheless, in the case of submicrometric Li metal nucleation, such as mossy/dendritic lithium, this contrast will not be observed owing to the small size of such particles for which the electromagnetic field penetrates the whole metal. As reported in the literature, during electrochemical cycling, an intense and symmetric EPR spectrum can appear. This feature is the manifestation of an alteration of the metallic anode surface that creates submicrometric lithium aggregates. However, in the present work, we did not perform operando measurements, and we did not observe an EPR signature of submicrometric lithium aggregates. Furthermore, in Fig. [Fig Ch1.F3]a, an intense semicircular shape localized at x=-6 mm and z=-6 mm, corresponding to the highest amplitude, can be observed. This result indicates a local defect much more sensitive to the microwave field that corresponds to the interface between the lithium electrode and the copper current collector. Finally, images in the y – z and y – x planes provide evidence that battery alignment inside the microwave loop-gap cavity is essential to reduce errors and misinterpretations. It can be seen that our solid-state pouch cell is slightly tilted in the loop gap, which may also explain the apparent amplitude variation. The semicircular shape is also clearly visible at x=-6 mm and y=-3 mm in the x – y plane but not in the y – z plane, indicating that the pouch cell is aligned in the x direction. Nevertheless, a three-axis field gradient associated with an L-band EPR spectrometer will enable us to locate metallic lithium structures through the standard packaging for batteries with a better spatial accuracy and will, therefore, improve the knowledge of Li-metal nucleation processes.

## 4 Conclusions

In conclusion, NMC ∥ Li^0^- and NMC ∥ Si-based solid-state pouch cell batteries have been studied by continuous-wave (cw) L-band EPR spectroscopy and imaging. We provide the first evidence of the EPR characterization of pouch cell batteries using standard aluminum-laminated packaging. We observed a metallic lithium EPR spectrum and a SiO_2_ defect signal through the aluminum-laminated film. An advantage of conventional cw-EPR spectroscopy at L-band is that a centimeter-scale pouch cell using an aluminum-laminated film material can be analyzed without preparation, without employing a home-made cell specially designed for EPR, and without opening the electrochemical battery.

For future studies, an operando EPR analysis of standard pouch cell batteries for solid-state or Li-ion technologies will provide additional information about the degradation processes and redox evolution by probing electrode materials directly through a standard aluminum-laminated film forming the pouch cell. We think that our new approach will provide valuable insight for the battery community in terms of the formulation, optimization, and quality control of standard and commercial batteries.

## Data Availability

The dataset that support the findings of this investigation is available from Zenodo: 10.5281/zenodo.14034164 [Bibr bib1.bibx4].

## Code availability

MATLAB is a commercial software from MathWorks, and the EasySpin package is available from https://easyspin.org/ [Bibr bib1.bibx7].
